# Isomangiferin promotes the migration and osteogenic differentiation of rat bone marrow mesenchymal stem cells

**DOI:** 10.1515/biol-2022-0884

**Published:** 2024-07-20

**Authors:** Bingjun Gao, Xin Cheng, Yarong Wu, Boyi Jiang

**Affiliations:** Department of Osteology, The People’s Hospital of Danyang, Affiliated Danyang Hospital of Nantong University, No. 2 Xinmin West Road, Danyang, Jiangsu, 212300, China; Department of Osteology, The People’s Hospital of Danyang, Affiliated Danyang Hospital of Nantong University, Danyang, Jiangsu, 212300, China

**Keywords:** failed bone healing, BMSCs, isomangiferin, osteogenic differentiation, AMPK/ACC pathway

## Abstract

Delayed or failed bone healing is a significant clinical challenge worldwide. Bone marrow mesenchymal stem cells (BMSCs) offer a promising approach for improving fracture healing. Isomangiferin, a xanthone C-glucoside, is known for its pharmacological activities, but its role in fracture healing remains unclear. In this study, we investigated the effects of isomangiferin on BMSCs under oxidative stress conditions induced by hydrogen peroxide (H_2_O_2_). Our results showed that isomangiferin promotes osteogenic differentiation and migration of H_2_O_2_-treated BMSCs, reduces apoptosis and reactive oxygen species production, and activates the AMP-activated protein kinase/acetyl-CoA carboxylase (AMPK/ACC) pathway. These findings suggest that isomangiferin may be a potential therapeutic agent for enhancing bone healing by modulating BMSC function.

## 1 Introduction

Delayed or failed bone healing is a significant clinical challenge worldwide, with approximately 5–10% of fractures resulting in delayed or non-union [1]. Bone marrow mesenchymal stem cells (BMSCs) have been reported to offer a promising method for accelerating fracture healing. Recent studies have shown encouraging results suggesting that BMSCs may promote fracture healing [2]. The ability of BMSCs to migrate to the fracture site, provide antioxidant protection, and exhibit osteogenic differentiation plays an important role in fracture healing [3]. After fracture, the migration of endogenous bone marrow mesenchymal stem cells to the fracture site is a key step in osteoblast maturation and mineralized tissue formation [3]. BMSCs migrate to the fracture site and then differentiate into osteoblasts and chondroblasts, promoting fracture healing through intramembranous ossification or intrachondral ossification [4]. Hypoxia caused by fracture and vascular injury triggers the expression of BMP-2 in BMSC, which initiates fracture healing [1]. To ameliorate the symptoms of bone healing failure, new and more effective treatments still need to be developed.

Traditional Chinese medicine has been widely used in clinical treatment of bone diseases in China for thousands of years. The natural product has a long history of use in traditional Chinese medicine to treat joint pain, hematochezia, hot flushes, and night sweats [5]. Isomangiferin, a xanthone C-glucoside, is one of the main components of the Cyclopia plant, a member of the Fabaceae family, known for its various pharmacological activities [6]. Isomangiferin provides protection against kidney injury in diabetic mouse models by inhibiting High Mobility Group Box 1 (HMGB1)/NOD-like Receptor Family Pyrin Domain Containing 3 (NLRP3)/NF-κB signaling [6]. In addition, isomangiferin exerts anti-breast cancer effect through functional inhibition of Vascular Endothelial Growth Factor Receptor 2 (VEGFR-2) [7]. Isomangiferin may be an effective treatment strategy for breast cancer by targeting VEGFR-2 [7]. The antiviral effects of isomangiferin may be due to their ability to inhibit intracellular viral replication [8]. However, the role and mechanism of isomangiferin in fracture healing are still unclear and need further study.

This study aimed to explore the effects of Isomangiferin on the bone healing *in vitro*. The results indicated that isomangiferin promotes BMSC motility and osteogenic differentiation through AMP-activated protein kinase/acetyl-CoA carboxylase (AMPK/ACC) pathway.

## 2 Materials and methods

### 2.1 Cell culture and treatment

Mouse BMSC line was bought from iCell Bioscience Inc (China) and maintained in Dulbecco's modified eagle medium (DMEM) with 10% Fetal bovine serum (FBS) at 37°C with 5% CO_2_. Cells were stimulated with 100 μM H_2_O_2_ (bought from Sigma) for 24 h. BMSCs were seeded at a density of 1 × 10^4^ cells per well in a 96-well plate. BMSCs were stimulated with isomangiferin (bought from Sigma) at the concentrations of 0, 2.5, 5, and 10 μM for 24 h. After 24 h of treatment with 100 µM H_2_O_2_, BMSCs were washed with PBS to remove residual H_2_O_2_, followed by treatment with isomangiferin. To evaluate whether the osteogenic differentiation induced by isomangiferin is reversible, BMSCs were washed with PBS after isomangiferin treatment and cultured for an additional 7 days before assessing osteogenic markers.

### 2.2 Cell viability

BMSC viability was detected by adding a cell counting kit-8 (CCK-8) solution. After treatment with H_2_O_2_ or isomangiferin, a CCK-8 agent was added to cells following washing with PBS. The OD450 value in each well was measured.

### 2.3 Transwell assays

200 µL of the cell suspension was added to the upper chamber of a Transwell insert (BD, 8 µm pore size). The lower chamber was filled with 600 µL of DMEM containing 10% FBS as a chemoattractant. Isomangiferin was added to the upper chamber and incubated to evaluate its effects on BMSC migration. After incubation, cells were fixed with 4% paraformaldehyde for 30 min and stained with crystal violet for 30 min.

### 2.4 Enzyme-linked immunosorbent assay (ELISA)

After indicated stimulations, cell supernatants were subjected to ELISA to determine the level of reactive oxygen species (ROS) (ab287839; Abcam) and ALP (ab285274; Abcam) following the manufacturer’s guidelines. For the determination of cytokines, refer to the methodology described in other works [9].

### 2.5 Alizarin Red staining

Alizarin Red staining was performed to assess mineralization. BMSCs were fixed with 4% formalin, permeabilized with PBS containing 0.1% Triton X-100, and incubated for 10 min with Alizarin Red staining solution (Beyotime, Beijing, China). The presence of bright red-orange staining indicates the deposition of calcium, a hallmark of mineralized extracellular matrix produced by osteoblasts. The stained samples were analyzed using an LSM710 microscope (Carl Zeiss, Germany). Images were captured and quantified using ImageJ to evaluate the degree of mineralization.

### 2.6 Cell apoptosis

For the detection of apoptotic cell number, Annexin V/Propyl iodide (PI) apoptosis detection was conducted following the manufacturer’s protocol (Sigma Aldrich, USA). 100 µL of the cell suspension was transferred to a flow cytometry tube, and 5 µL of Annexin V-FITC and 5 µL of PI were added. The cells were gently vortexed and incubated for 15 min at room temperature in the dark. After incubation, 400 µL of 1× binding buffer was added to each tube, and the samples were analyzed by flow cytometry within 1 h. A total of 10,000 cells per sample were analyzed using a BD LSRFortessa™ X-20 flow cytometer (BD, USA). The percentage of apoptotic cells was determined by analyzing the Annexin V-FITC and PI fluorescence signals, with early apoptotic cells being Annexin V positive and PI negative, and late apoptotic or necrotic cells being Annexin V positive and PI positive [9].

### 2.7 Immunoblot assay

Proteins were separated by 10% Sodium dodecyl sulfate polyacrylamide gel electrophoresis and transferred onto PVDF membranes, followed by blocking with 5% BSA in TBST buffer. Subsequently, membranes were conjugated with primary antibodies targeting Bcl-2-associated X protein (Bax) (Abcam, ab32503; 1:1,000), B-cell lymphoma 2 (Bcl-2) (Abcam, ab182858; 1:1,000), Cleaved caspase-3 (Abcam, ab32042; 1:1,000), Matrix Metalloproteinase 2 (MMP-2) (Abcam, ab92536; 1:1,000), MMP-9 (Abcam, ab76003; 1:1,000), Runx2 (Abcam, ab192256; 1:1,000), Bone Morphogenetic Protein 2 (BMP2) (Abcam, ab284387; 1:1,000), ACC (Invitrogen, MA5-15025; 1:1,000), p-ACC (Ser79, Invitrogen, PA5-17725; 1:500), MAPK (Abcam, ab2047; 1:1,000), p-MAPK (Abcam, ab133448; 1:500), and GAPDH (Abcam, ab8245, 1:3,000) for 1 h. Subsequently, the membranes were incubated with specific secondary antibodies for 1 h and the blots were analyzed with an ECL kit. The methodology for this analysis was adapted from a previous study [10].

### 2.8 Statistics

Statistical analysis was performed using GraphPad Prism 5.0 software. Data were represented as mean ± SD. One-way ANOVA for multiple groups was used to determine statistical significance. A *p*-value of <0.05 was considered statistically significant. All experiments were repeated three times independently.

## 3 Results

### 3.1 Isomangiferin contributes to the osteogenic differentiation of H_2_O_2_-stimulated BMSCs

**Figure 1:**
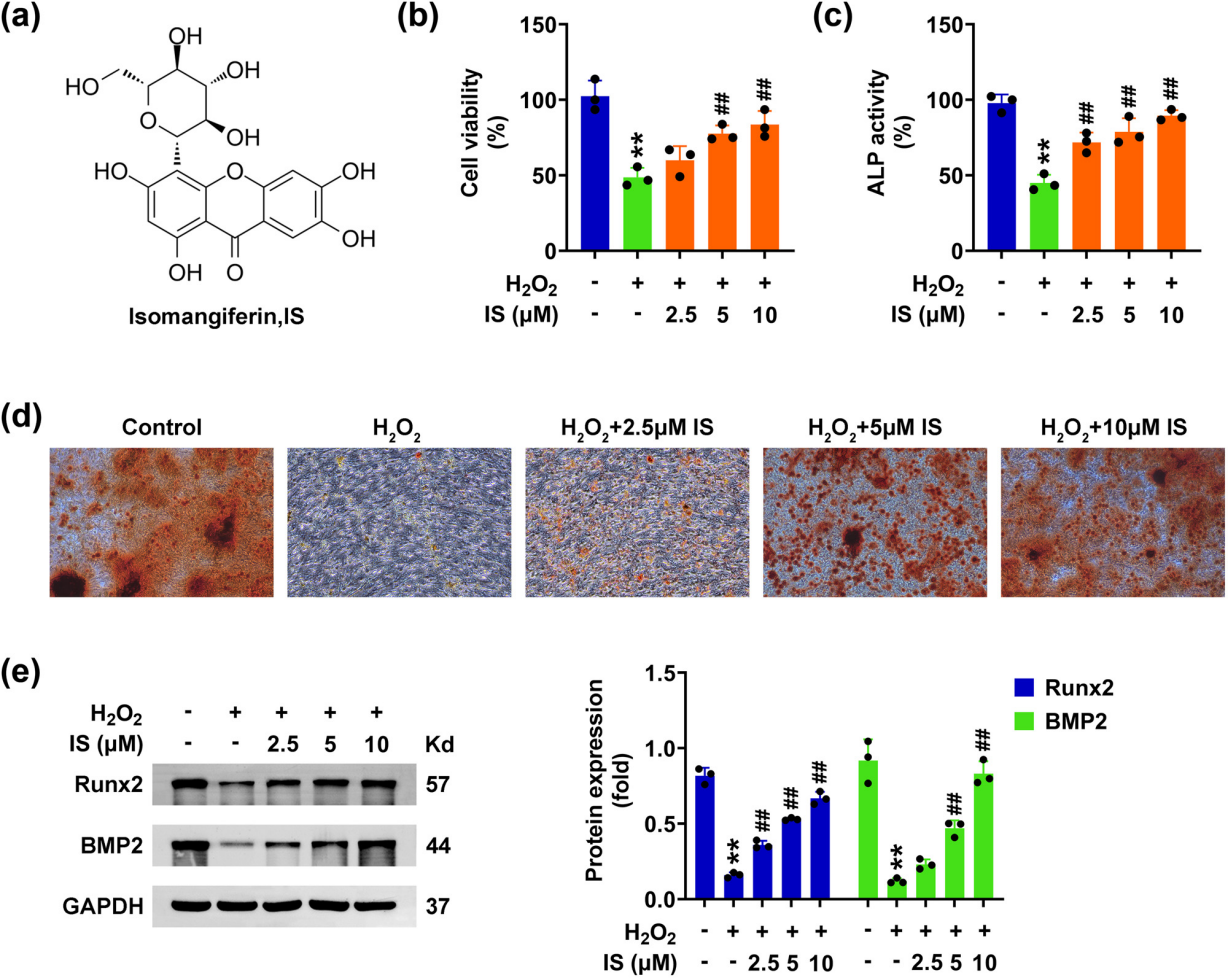
Isomangiferin contributes to the osteogenic differentiation of H_2_O_2_-stimulated BMSCs. (a) Molecular formula of isomangiferin. (b) CCK-8 assays showed the effects of isomangiferin (2.5, 5, 10 μM) on H_2_O_2_-stimulated BMSCs for 24 h. The OD450 value was measured. (c) ALP activity detection showed the ALP activity in BMSCs upon the treatment of isomangiferin (2.5, 5, 10 μM) and 100 μM H_2_O_2_ for 24 h. (d) Alizarin Red staining showed the osteogenic differentiation degree of BMSCs upon the treatment of isomangiferin (2.5, 5, 10 μM) and 100 μM H_2_O_2_ for 24 h. (e) Immunoblot showed the expression of Runx2 and BMP2 in BMSCs upon the treatment of isomangiferin (2.5, 5, 10 μM) and 100 μM H_2_O_2_ for 24 h. **p < 0.01, H_2_O_2_ vs control, ^##^ *p* < 0.01, \[{\text{H}}_{\text{2}}{\text{O}}_{2}^{+}]\] isomangiferin, vs H_2_O_2_. IS, isomangiferin.

To detect the effects of isomangiferin on bone healing, particularly the effects on BMSCs, a failed bone healing cell model was constructed using H_2_O_2_ to treat BMSCs. The molecular formula of isomangiferin is shown in Figure 1a. CCK-8 assays indicated that H_2_O_2_ significantly decreased the growth of BMSCs (Figure 1b), whereas isomangiferin promoted the growth of H_2_O_2_-stimulated BMSCs, with the increased OD450 value (Figure 1b). Consistently, the ALP activity was detected by the kit, which reflected the osteogenic differentiation capacity. The data indicated that H_2_O_2_ significantly decreased ALP activity, whereas isomangiferin increased the ALP activity of H_2_O_2_-stimulated BMSCs, suggesting the promotion of osteogenic differentiation (Figure 1c). Similarly, Alizarin red staining exhibited that H_2_O_2_ significantly blocked osteogenic differentiation (Figure 1d). However, isomangiferin promoted theosteogenic differentiation of H_2_O_2_-stimulated BMSCs (Figure 1d). Immunoblot assays further indicated that H_2_O_2_ significantly decreased the expression of osteogenic differentiation marker Runt-related Transcription Factor 2 (Runx2) and BMP2, whereas isomangiferin promoted the expression of these factors (Figure 1e). Collectively, isomangiferin contributes to the osteogenic differentiation of H_2_O_2_-stimulated BMSCs.

### 3.2 Isomangiferin promotes the migration of H_2_O_2_-stimulated BMSCs

**Figure 2:**
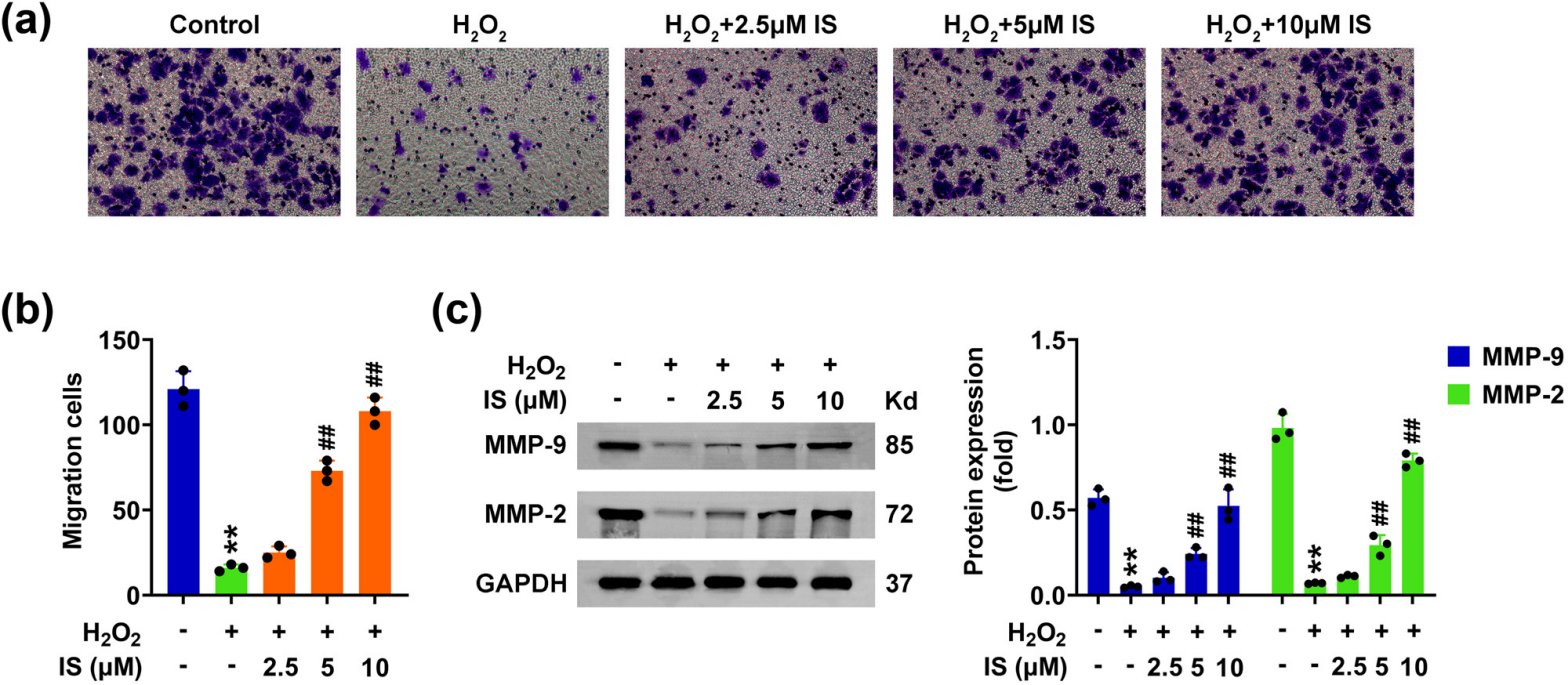
Isomangiferin promotes the migration of H_2_O_2_-stimulated BMSCs. (a) Transwell assays showed the invasion levels of BMSCs upon the treatment of isomangiferin (2.5, 5, 10 μM) and 100 μM H_2_O_2_ for 24 h. (b) Quantification of panel (a). The migration cell numbers per field were quantified. (c) Immunoblot showed the expression of MMP-2 and MMP-9 in BMSCs upon the treatment of isomangiferin (2.5, 5, 10 μM) and 100 μM H_2_O_2_ for 24 h. **p < 0.01, H_2_O_2_ vs control, ^##^ *p* < 0.01, \[{\text{H}}_{\text{2}}{\text{O}}_{2}^{+}]\] isomangiferin, vs H_2_O_2_. IS, isomangiferin.

Subsequently, the effects of isomangiferin on the migration of H_2_O_2_-stimulated BMSCs were detected. Transwell assays showed that H_2_O_2_ decreased the migrated cell numbers of BMSCs (Figure 2a). However, isomangiferin reversed the suppression of cell migration in BMSCs caused by H_2_O_2_ treatment (Figure 2a). Similarly, immunoblot assays confirmed that H_2_O_2_ treatment decreased the expression of MMP-2 and MMP-2, two migration markers (Figure 2b), whereas isomangiferin treatment increased the expression of these markers in H_2_O_2_-stimulated BMSCs, suggesting the promoting of migration (Figure 2b). Therefore, isomangiferin promotes the migration of H_2_O_2_-stimulated BMSCs.

### 3.3 Isomangiferin further restrains the apoptosis and ROS production in H_2_O_2_-stimulated BMSCs

**Figure 3:**
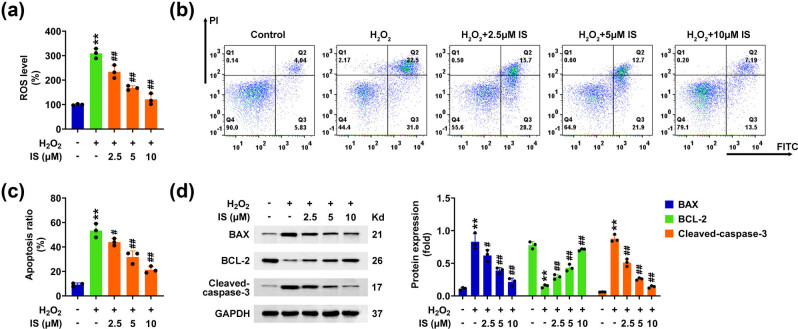
Isomangiferin further restrains the apoptosis and ROS production in H_2_O_2_-stimulated BMSCs. (a) ROS levels in BMSCs upon the treatment of isomangiferin (2.5, 5, 10 μM) and 100 μM H_2_O_2_ for 24 h. (b) Flow cytometric assays showed the apoptosis degree of BMSCs upon the treatment of isomangiferin (2.5, 5, 10 μM) and 100 μM H_2_O_2_ for 24 h. (c) Quantification of panel (b). The percentage of apoptosis cells was quantified. (d) Immunoblot showed the expression of BAX, Bcl-2, and cleaved caspase-3 in BMSCs upon the treatment of isomangiferin (2.5, 5, 10 μM) and 100 μM H_2_O_2_ for 24 h. **p < 0.01, H_2_O_2_ vs control, ^#^ *p* < 0.05, ^##^ *p* < 0.01, \[{\text{H}}_{\text{2}}{\text{O}}_{2}^{+}]\] isomangiferin, vs H_2_O_2_. IS, isomangiferin.

Next, the effects of isomangiferin on the apoptosis and ROS production of BMSCs were investigated. It was noticed that H_2_O_2_ treatment significantly increased ROS production in BMSCs, whereas isomangiferin treatment decreased ROS production in H_2_O_2_-stimulated BMSCs (Figure 3a). Flow cytometry analysis indicated that H_2_O_2_ stimulated the apoptosis of BMSCs with an increased percentage of apoptosis cells (Figure 3b and c). However, isomangiferin treatment restrained the apoptosis of H_2_O_2_-stimulated BMSCs (Figure 3b and c). Consistently, Immunoblot assays showed that H_2_O_2_ contributed to the expression of apoptosis markers, including BAX and cleaved caspase-3, and decreased Bcl-2 expression, whereas isomangiferin reversed the expression of these factors caused by H_2_O_2_ treatment in BMSCs (Figure 3d). Therefore, isomangiferin further restrains the apoptosis and ROS production in H_2_O_2_-stimulated BMSCs.

### 3.4 Isomangiferin contributes to AMPK/ACC pathway in H_2_O_2_-stimulated BMSCs

**Figure 4:**
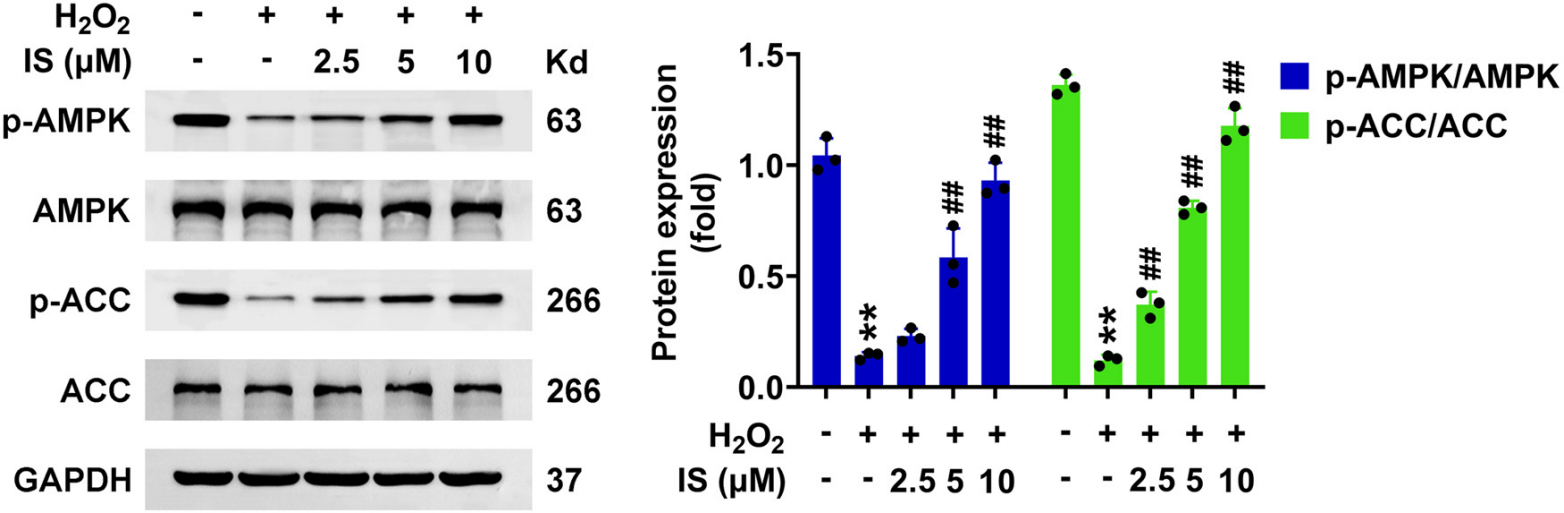
Isomangiferin contributes to AMPK/ACC pathway in H_2_O_2_-stimulated BMSCs. Immunoblot showed the expression and phosphorylation levels of AMPK and ACC in BMSCs upon the treatment of isomangiferin (2.5, 5, 10 μM) and 100 μM H_2_O_2_ for 24 h. **p < 0.01, H_2_O_2_ vs control, ^##^ *p* < 0.01, \[{\text{H}}_{\text{2}}{\text{O}}_{2}^{+}]\] isomangiferin, vs H_2_O_2_. IS, isomangiferin.

Finally, the potential mechanism was explored. AMPK/ACC pathway was reported to affect the activity of BMSCs, such as osteogenic differentiation and migration, and therefore the Immunoblot was conducted. The results indicated that H_2_O_2_ treatment decreased the phosphorylation of AMPK and ACC in BMSCs (Figure 4). However, isomangiferin incubation increased the phosphorylation of AMPK and ACC in H_2_O_2_-stimulated BMSCs, suggesting the activation of this pathway (Figure 4). Therefore, isomangiferin contributes to the AMPK/ACC pathway in H_2_O_2_-stimulated BMSCs.

## 4 Discussion

Bone healing failure, also known as nonunion, is a medical condition where a fractured bone fails to heal properly [8]. This can be a serious complication after a bone fracture and can result in chronic pain, swelling, and sometimes disability. The causes of bone healing failure may vary, but common factors include poor blood supply to the bone, severe trauma, infection, or inadequate stabilization of the fracture [8]. Treatment for nonunion typically involves addressing the underlying cause. This can include surgical interventions to stabilize the bone, grafting procedures to provide new bone material, and the use of specialized devices such as bone stimulators that promote healing. The treatment of bone healing failure with traditional Chinese medicine mainly relies on traditional herbs and therapeutic methods to promote bone healing and overall health [11,12]. Commonly used herbs include *Psoralea corylifolia*, *Rehmannia glutinosa*, *Cinnamomum cassia*, and *Achyranthes bidentata*, which are believed to help strengthen bones, improve blood circulation, and fortify muscles [13,14]. Herein, our results indicated that isomangiferin promotes migration and osteogenic differentiation of BMSCs through the AMPK/ACC pathway. Therefore, isomangiferin has the potential to serve as a promising drug for bone healing failure.

In this study, we found that isomangiferin promotes the motility and osteogenic differentiation of BMSCs through the AMPK/ACC pathway. These results are consistent with previous research that has highlighted the osteogenic potential of isomangiferin in various models. Additionally, the anti-inflammatory and antioxidant properties of isomangiferin reported in other studies complement our findings, suggesting a multifaceted role of isomangiferin in bone healing. Comparing our results with other works, it is evident that isomangiferin consistently exhibits beneficial effects across different models, further reinforcing its potential as a therapeutic agent for bone healing. However, the specific pathways and mechanisms may vary depending on the model and experimental conditions, highlighting the need for further research to fully understand the role of isomangiferin in bone health.

BMSCs play a crucial role in bone healing. These multipotent stem cells can differentiate into bone cells, cartilage cells, and more, essential for new bone tissue formation [15]. BMSCs promote bone regeneration by secreting growth factors, regulate immune responses to reduce inflammation, and support angiogenesis, which are important aspects of bone healing [16]. Consequently, BMSCs are extensively studied in regenerative medicine and tissue engineering, especially in enhancing the repair of bone fractures and defects [16]. However, their clinical application still faces several challenges, such as determining cell sources, improving culture techniques, and evaluating long-term safety and effectiveness. Herein, our results indicated that isomangiferin promotes osteogenic differentiation and migration of BMSCs, and reduces intracellular ROS formation and apoptosis. Therefore, it could affect bone healing by mediating BMSC function.

Isomangiferin, a natural flavonoid compound with a range of biological activities [7]. It is known for its potent antioxidant properties, neutralizing free radicals and reducing oxidative stress [6,7,17]. Additionally, it exerts anti-inflammatory effects by reducing the production of inflammatory mediators [6]. Isomangiferin also possesses antimicrobial and antiviral activities, inhibiting certain bacteria and viruses [6]. Preliminary studies suggest its potential in cancer treatment by inducing apoptosis and inhibiting cell proliferation of cancer cells [7]. Furthermore, it shows promise in managing diabetes by regulating blood glucose levels and improving symptoms [17]. The data in this study indicated its effects on the osteogenic differentiation, migration, and ROS production of BSMCs. However, more studies are needed to fully understand its mechanisms and validate these effects for medical applications. It is advisable to consult healthcare professionals before using any supplements or treatments based on isomangiferin.

The AMPK/ACC pathway has been reported to play a significant role in the regulation of BMSC activity, including osteogenic differentiation and migration [18]. Given the importance of this pathway in BMSC function and its potential involvement in bone healing, we were particularly interested in investigating whether isomangiferin exerts its effects on BMSCs through the modulation of the AMPK/ACC pathway. Previous studies have suggested that activation of the AMPK/ACC pathway can enhance the osteogenic potential of BMSCs and promote bone regeneration [18]. AMPK, an energy-sensing kinase, promotes the differentiation of bone precursor cells when energy levels are low, contributing to new bone formation [19]. By regulating ACC, which is crucial for fatty acid synthesis, AMPK affects cellular energy metabolism and indirectly affects bone metabolism and repair. Activation of AMPK enhances the function of osteoblasts, accelerating fracture healing [19,20]. The current study focuses on how the AMPK/ACC pathway can be manipulated to control bone healing, which holds potential significance for developing new treatments for fractures and osteoporosis [19,20]. Herein, our results suggest that isomangiferin promotes the AMPK/ACC pathway and therefore contributes to bone healing. However, the precise mechanism needs further study.

In the remodeling of bone tissue, cytokines play a crucial role as mediators and stimulators of various processes [18]. They are involved in the regulation of bone resorption and formation, acting as key signaling molecules that influence the activity of osteoclasts and osteoblasts. Additionally, cytokines are involved in the inflammatory response associated with bone remodeling, where they can stimulate necrosis and fibrosis, further impacting the bone healing process [19]. Understanding the role of cytokines in bone remodeling is essential for developing targeted therapies for bone-related disorders.

In summary, isomangiferin promotes BMSC motility and osteogenic differentiation through the AMPK/ACC pathway.
